# Detecting Linkage between a Trait and a Marker in a Random Mating Population without Pedigree Record

**DOI:** 10.1371/journal.pone.0004956

**Published:** 2009-03-24

**Authors:** Shuhei Mano, Takaho A. Endo, Akira Oka, Akira Ozawa, Takashi Gojobori, Hidetoshi Inoko

**Affiliations:** 1 Graduate School of Natural Sciences, Nagoya City University, Nagoya, Japan; 2 Japan Science and Technology Agency, Saitama, Japan; 3 RIKEN Genomic Science Center, Kanagawa, Japan; 4 Department of Molecular Life Science, Tokai University School of Medicine, Kanagawa, Japan; 5 Department of Dermatology, Tokai University School of Medicine, Kanagawa, Japan; 6 Center for Information Biology and DNA Data Bank of Japan, National Institute of Genetics, Mishima, Japan; Institute of Infectious Disease and Molecular Medicine, South Africa

## Abstract

Modern linkage-based approaches employing extended pedigrees are becoming powerful tools for localizing complex quantitative trait loci. For these linkage mapping methods, it is necessary to reconstruct extended pedigrees which include living individuals, using extensive pedigree records. Unfortunately, such records are not always easy to obtain and application of the linkage-based approaches has been restricted. Within a finite population under random mating, latent inbreeding rather than non-random inbreeding by consanguineous marriages is expected to occur and is attributable to coalescence in a finite population. Interestingly, it has been revealed that significant random inbreeding exists even in general human populations. Random inbreeding should be used to detect the hidden coancestry between individuals for a particular chromosomal position and it could also have application in linkage mapping methods. Here we present a novel method, named finite population based linkage mapping (FPL) method, to detect linkage between a quantitative trait and a marker via random inbreeding in a finite population without pedigree records. We show how to estimate coancestry for a chromosomal position between individuals by using multipoint Bayesian estimation. Subsequently, we describe the FPL method for detecting linkage via interval mapping method using a nonparametric test. We show that the FPL method does work via simulated data. For a random sample from a finite population, the FPL method is more powerful than a standard pedigree-based linkage mapping method with using genotypes of all parents of the sample. In addition, the FPL method was demonstrated by actual microsatellite genotype data of 750 Japanese individuals that are unrelated according to pedigree records to map a known Psoriasis susceptible locus. For samples without pedigree records, it was suggested that the FPL method require limited number of individuals, therefore would be better than other methods using thousands of individuals.

## Introduction

Identifying susceptible loci of complex common disease is an important challenge in human statistical genetics. Whole genome association scans among unrelated individuals is a popular tool for localizing susceptible genes of complex common diseases. The crux upon which association-based approaches rests is the common disease common variant hypothesis. In cases where this hypothesis does not hold true, e.g. a disease caused by multiple rare variants that have small effects, association-based approaches may lose their power [1], [2]. Therefore, linkage-based approaches could provide useful complements to association-based approaches [3].

Many complex common diseases are evaluated and diagnosed directly on a quantitative scale. Modern linkage-based approaches employing extended pedigrees are becoming powerful tools for localizing quantitative trait loci, and they had led to the successful identification of human quantitative trait loci [3]. In these linkage mapping methods, however, we must reconstruct extended pedigrees that include living individuals, because we need prior information concerning the sharing of homologous genes by identical by descent (IBD) among pedigree members. The application of these approaches has been restricted to cases where accurate pedigree records are available.

Sewall Wright introduced F statistics, which measure degree of coancestry between pairs of homologous genes within a finite population [4]–[6]; 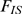 represents the probability that two homologous genes of a single individual are descended from the same gene in a common ancestor, namely, IBD, while 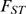 is the probability that two homologous genes, chosen at random from a population, are IBD. He emphasized that 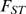 is caused by random drift of gene frequency within a finite population (rather than by departures from Hardy-Weinberg ratios within the population) and is irreversible (except by mutation, selection, or migration). 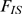, on the other hand, is caused by consanguineous marriages, and immediately becomes zero with random mating. Allen called 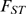 and 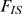 random and nonrandom inbreeding, respectively [5]. Interestingly, recent analyses of whole genome single nucleotide polymorphisms (SNPs) data analysis revealed the value of 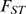 to be 0.13 for the whole human population [7]; this value is significantly higher than that had been estimated by isonymy in genetically isolated populations [5], [6].

Here, we propose a novel method, named finite population based linkage mapping (FPL) method, to detect linkage between a quantitative trait and a marker via random inbreeding in a finite population without pedigree records. Considering IBD status among four homologous genes of a pair of individuals, we devise a consistent population genetic model for random inbreeding. Then, using computer simulations assuming usage of microsatellite and SNPs data, we illustrate significance and power of the test. Finally, we demonstrate the linkage mapping method to the microsatellite genotype data of Japanese psoriasis patients and healthy controls that are unrelated according to pedigree records but are expected to be distantly related.

## Methods

### Coancestry within a finite Population under Random Mating

Consider a random mating population which consists of 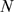 diploid individuals founded 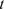 generations ago. Assume we have genotypes of 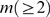 multiallelic markers of 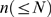 individuals randomly sampled from a population, since it is not always possible to sample all individuals from a population. Let the genotypes of the *j*-th marker of the *i*-th pair of individuals be 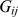, where 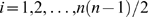; 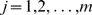, and the absolute differences between the trait values for the *i*-th pair of individuals be 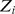.

**Figure 1:**
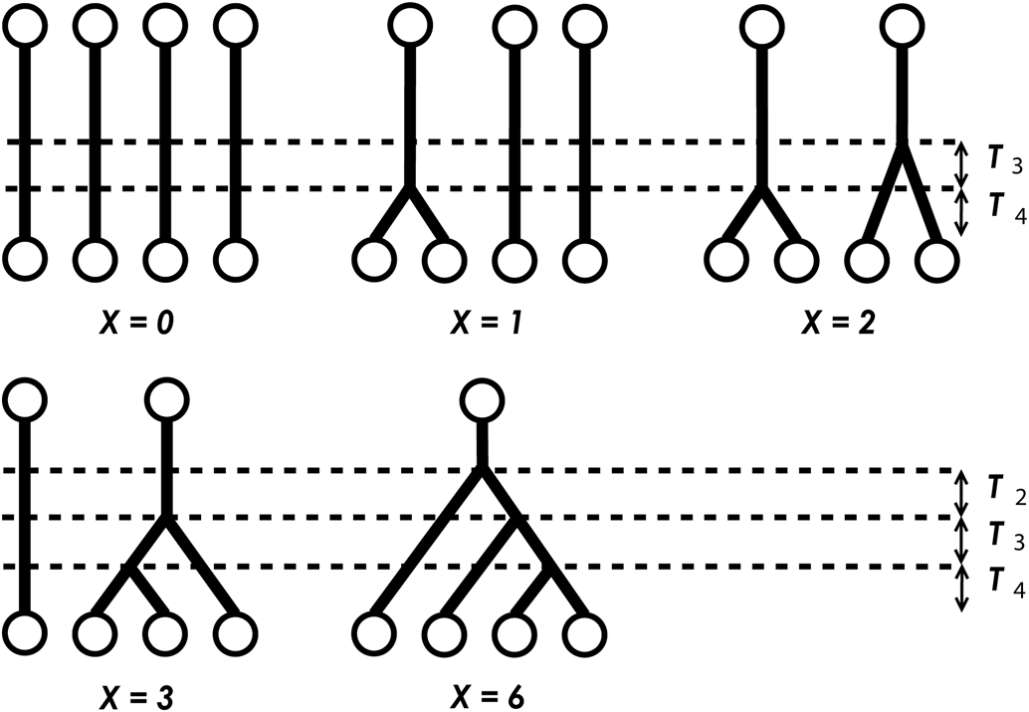
Coalescence events among four alleles randomly sampled from a population. 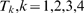
 is the time to the most recent common ancestor of 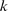 alleles randomly sampled from a population. 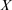 is the number of IBD pairs of alleles when choosing two alleles randomly from the union of four alleles of a marker of a pair of individuals. For simplicity, subscripts 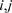 are omitted.

**Figure 2:**
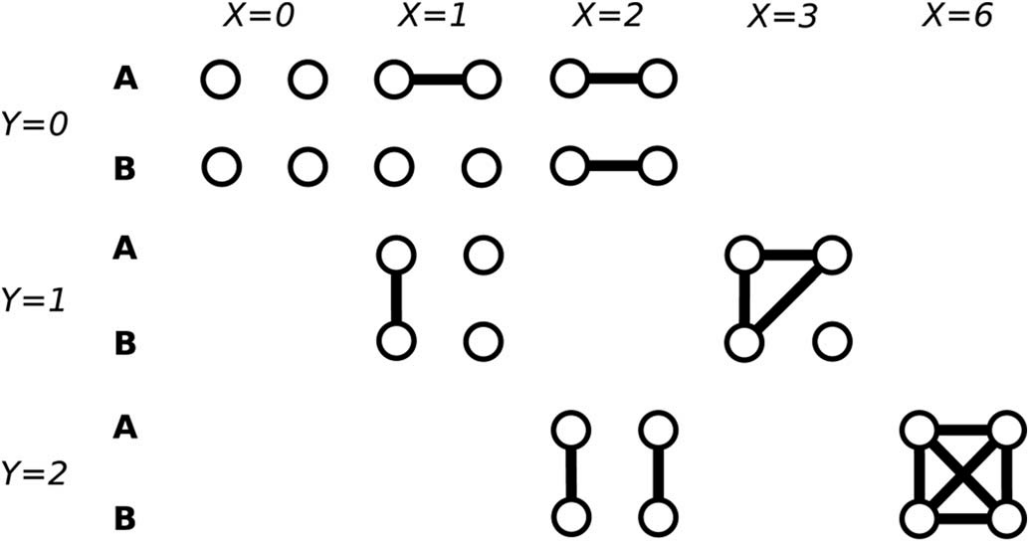
Shared alleles between individuals A and B by coalescence events. IBD alleles are connected by lines. 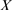 is the number of IBD pairs of alleles when choosing two alleles randomly from the union of four alleles of a marker of a pair of individuals. 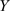 is the number of shared alleles of a marker between a pair of individuals by IBD. For simplicity, subscripts 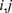 are omitted.

Let us now introduce a random variable 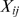, 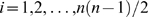; 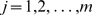, which is the number of IBD pairs of alleles when choosing two alleles randomly from the union of four alleles of the *j*-th marker of the *i*-th pair of individuals (Figure 1). Also, introduce a random variable 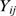, 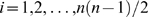; 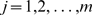,, which is the number of shared alleles between the *j*-th marker of the *i*-th pair of individuals by IBD (Figure 2). The unconditional probability distributions of 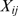 and 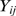, which are the prior distributions in the Bayesian terminology, are determined solely by 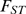 (see Appendix S1).

The most general means of describing the relatedness of one individual to another is in terms of the nine IBD modes, the condensed coefficients of identity, which are denoted as 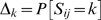 [8], [9] (see Chapter 7 in [10]), where 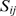 is the IBD mode. Note that 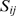 has close relationship with 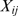 and 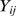. The event 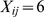, 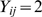 is identical to 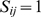, the event 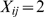, 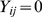 is identical to the event 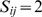, the event 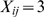, 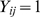 is identical to a union of the events 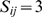 and 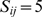, the event 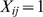, 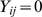 is identical to a union of the events 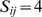 and 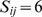, the event 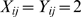 is identical to the event 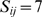, the event 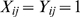 is identical to the event 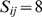, and the event 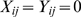 is identical to the event 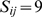. In an infinite population only 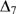, 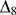 and 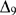 are nonzero, while in a finite population all coefficients could be nonzero.

Table S1 gives the conditional probability distribution of 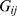, when the IBD mode (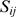) is given. We have (see Appendix S1 for the derivation)
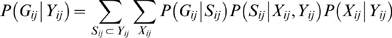
(1)Note that 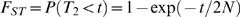, where 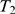 is the coalescence time of two alleles taken randomly from the population (see Section 9.2 in [11]). We estimate the allele frequencies using the sample. Further, we estimate 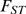 by homozygosity, as proposed by [6]. By using a coalescence argument, it is straightforward to show that homozygosity is a moment estimator of 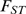.

### Detecting Linkage

The chain of 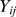, 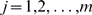 along the four chromosomes of the the *i*-th pair of individuals can be modeled by a three-state hidden Markov model. By using the hidden Markov model, it is possible to compute the multipoint posterior probability distribution 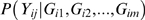 (see Appendix S1 for details). In a random mating population, the conditional expectation of 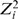 when 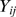 is given is 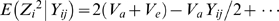, where 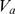 and 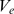 are the additive and the environmental variance of the trait, respectively (see Appendix S1 for the derivation). Thus, linkage between the trait and the *j*-th marker can be detected by a regression, which is a regression of 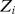 onto the multipoint posterior estimates of 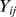. The standard parametric regression is anti-conservative because of correlations among observations 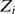, however, the Mantel test can overcome this difficulty (see Appendix S1 for details). Moreover, an interval mapping method can give the multipoint posterior estimate at any points on a map (see Appendix S1 for details). In the present paper, we examine the interval mapping method.

### Simulation Setup

We generated random mating populations founded 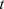 generations ago, which consisted of 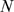 diploid individuals. Throughout the present paper, four cases of population demography will be examined, unless otherwise stated; Case 1: 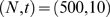, Case 2: (500,100), Case 3 : (5000,100), and Case 4: (5000,1000). 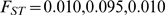, and 0.095 for Case 1–4, respectively. We assumed the usage of microsatellite markers, since evenly spaced microsatellite markers are widely used for linkage scans. The stepwise mutation model [12] was assumed for each marker with a mutation rate of 0.0001. We generated the genotypes of 10 evenly spaced markers. In the founder population, we set the markers to be evenly segregating. The trait locus was assumed to be at the center of the map (the mid-point of the 5-th and 6-th markers), in which two neutral alleles were evenly segregating. The allele frequencies in the present population were random variables, since they changed scholastically with random drift. Further, the usage of SNP markers was also assumed, since dense SNP markers are now becoming available. We generated the genotypes of 100 evenly spaced SNPs, in which two alleles are evenly segregating. In addition, to investigate the usage of SNP haplotypes, we regarded the 100 SNPs as 10 SNP haplotypes, each comprising of 10 contiguous SNPs, since tests by this choice were generally comparable or more powerful than those by other choices, including 20 haplotypes of 5 SNPs, 5 haplotypes of 20 SNPs, and 2 haplotypes of 50 SNPs). Linkage mapping was conducted by scoring an SNP haplotype as an allele.

### Experimental Methods

We determined genotypes of 8 microsatellite markers on the chromosome 6 of 375 Japanese patients with psoriasis vulgaris and 375 healthy controls. The study was approved by the local ethics committee, and informed consent was obtained from all the participants. Since no quantitative measures of disease were available for these individuals, we set the trait values as 1 for the patients and 0 for the controls. The 8 microsatellite markers were D6S942, F13A1, D6S2434, D6S1660, D6S2931 (also known as D6S2678, M6S168, and C1_4_4), D6S2427, D6S1017, and D6S2410, and their positions from the telomere in the Marshfield genetic map were 0, 11.11 25.08, 40.14, 46.81, 53.81, 63.28 and 73.1 in cM, respectively, with the positions of F13A1 and D6S2931 were estimated on the basis of their physical positions in the human genome (Mar. 2006, hg18). The genotypes of D6S2931 are provided in Table S2.

## Results

### Significance and Power

We investigated the anti-conservativeness of the standard parametric regression. We set the trait to be unlinked. For each case of population demography, 1,000 populations with microsatellite maps with 0, 1, 5, and 10 cM intervals. From each simulated population 500 individuals were randomly sampled, and p-values by the Mantel test and the t-test of the regression were computed. The proportion of populations for which the p-value was less than 0.05 is shown in Table 1. From the p-values determined, it is clear that the p-value of the t-test of the regression is anti-conservative, as expected. On the other hand, the Mantel test behaved regularly: the proportion of populations for which the p-values were less than 0.05 approximated the value expected in the absence of bias, regardless of the parameters used. Thus, in the following presentations, we will show the results of the Mantel test, unless otherwise stated. The t-test of the regression was generally more powerful and required a short computation time, however, its results should only be used as a reference.

**Table 1:** Significance of the tests.

Population	Test*	Marker Interval (cM)						
		Microsatellite				SNP		
		0	1	5	10	0.01	0.01H	0.1H
Case 1	M	0.048	0.048	0.045	0.044	0.050	0.055	0.043
	R	0.320	0.290	0.193	0.143	0.839	0.338	0.320
Case 2	M	0.057	0.054	0.065	0.050	0.056	0.047	0.046
	R	0.627	0.391	0.287	0.258	0.810	0.614	0.443
Case 3	M	0.045	0.054	0.051	0.063	0.045	0.045	0.034
	R	0.345	0.169	0.123	0.137	0.835	0.351	0.195
Case 4	M	0.063	0.051	0.054	0.046	0.053	0.051	0.046
	R	0.543	0.243	0.285	0.271	0.825	0.549	0.188

* M is the Mantel test and R is the t-test by the regression.

Standard pedigree-based linkage analysis is prone to large increase in type I errors when the markers are tightly linked and there is large linkage disequilibrium among them. It comes from assuming linkage equilibrium among markers in linkage disequilibrium when there is missing phase information [13]. In contrast, since the FPL method does not explicitly use founder phases, it is robust in preventing the inflation of type I errors caused by linkage disequilibrium among markers. In fact, as long as the Mantel test was used, the type I errors did not increase with the new linkage mapping method, even when the markers were completely linked (Table 1).

Subsequently, we investigated the power of the FPL method in various models of a trait locus. The broad sense heritability, proportion of the trait variance determined by the trait locus: 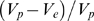, where 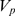 is the phenotypic variance (See Chapter 4 in [10]), was set to be 50%, and 10%, and the additive and completely dominant modes were assumed. For each case of population demography, 1,000 populations with microsatellite maps with 0, 1, 5, and 10 cM intervals are simulated. From each simulated population 500 individuals were randomly sampled and p-values were computed by using the Mantel test (in the cases that the population size is 500, all individuals were used). The proportion of populations for which the p-values was less than 0.05 at the trait locus is shown in Table 2 (for 50% heritability) and Table 3 (for 10% heritability). It was difficult to detect the trait locus with 10% heritability, even when completely linked markers were used. In other words, if a 1 cM map is already available, efforts to increase the marker density to gain more power would fail, since power would already saturated because of linkage disequilibrium among the markers. On the other hand, we could detect the trait locus with 50% heritability using markers with 1 cM intervals. In the population whose size is 500, even the markers with 10 cM intervals were enough to detect the trait locus with 50% heritability; however, this result implies that a finer resolution than 10 cM could not be obtained, because of linkage disequilibrium among the markers. We also investigated the improvement of the power by extreme sampling, where individuals who had the top 250 and bottom 250 values for the quantitative trait were sampled from the population. The results are also shown in Tables 2 and 3. The power improved considerably. The markers spaced with 5 cM intervals were satisfactory to detect the trait locus with 50% heritability.

**Table 2:** Power in various samplings for a trait locus with 50% heritability.

Trait*	Population	Marker Interval (cM)													
		Random Sampling							Extreme Sampling						
		Microsatellite				SNP**			Microsatellites				SNP		
		0	1	5	10	0.01	0.01H	0.1H	0	1	5	10	0.01	0.01H	0.1H
A	Case 1	1.00	1.00	1.00	1.00	0.08	1.00	1.00	—	—	—	—	—	—	—
	Case 2	1.00	1.00	1.00	0.96	0.86	1.00	1.00	—	—	—	—	—	—	—
	Case 3	1.00	1.00	0.49	0.18	0.10	1.00	1.00	1.00	1.00	1.00	0.81	0.61	1.00	1.00
	Case 4	1.00	0.94	0.21	0.16	0.78	1.00	1.00	1.00	0.94	0.62	1.00	1.00	1.00	1.00
D	Case 1	1.00	1.00	0.99	0.95	0.07	1.00	1.00	—	—	—	—	—	—	—
	Case 2	1.00	0.99	0.95	0.77	0.80	1.00	0.97	—	—	—	—	—	—	—
	Case 3	1.00	0.97	0.28	0.11	0.10	1.00	0.97	1.00	0.88	0.53	1.00	0.44	1.00	1.00
	Case 4	1.00	0.77	0.19	0.10	0.69	0.96	0.91	1.00	0.74	0.33	1.00	0.99	1.00	1.00

* A and D are the additive and the complete dominant mode, respectively.

** H is haplotype consists of 10 contiguous SNPs.

**Table 3:** Power in various samplings for a trait locus with 10% heritability.

Trait*	Population	Marker Interval (cM)													
		Random Sampling							Extreme Sampling						
		Microsatellite				SNP**			Microsatellites				SNP		
		0	1	5	10	0.01	0.01H	0.1H	0	1	5	10	0.01	0.01H	0.1H
A	Case 1	0.42	0.35	0.28	0.25	0.03	0.37	0.35	—	—	—	—	—	—	—
	Case 2	0.73	0.65	0.35	0.21	0.25	0.73	0.48	—	—	—	—	—	—	—
	Case 3	0.28	0.24	0.07	0.06	0.06	0.37	0.27	1.00	1.00	0.57	0.18	0.26	1.00	1.00
	Case 4	0.72	0.18	0.09	0.10	0.20	0.49	0.32	1.00	0.96	0.42	0.21	0.99	1.00	1.00
D	Case 1	0.28	0.25	0.16	0.19	0.03	0.24	0.21	—	—	—	—	—	—	—
	Case 2	0.54	0.40	0.26	0.21	0.18	0.39	0.33	—	—	—	—	—	—	—
	Case 3	0.17	0.15	0.07	0.06	0.04	0.24	0.14	1.00	0.95	0.25	0.10	0.14	1.00	0.99
	Case 4	0.45	0.16	0.09	0.05	0.16	0.26	0.18	1.00	0.77	0.19	0.10	0.95	1.00	0.99

* A and D are the additive and the complete dominant mode, respectively.

** H is haplotype consists of 10 contiguous SNPs.

In addition, we investigated the significance and power and of the new linkage mapping method by assuming the usage of SNP markers. SNP maps of 0.01 and 0.1 cM interval were generated for each parameter set, the models and the samplings. One of the strategies employed involved the direct use of these SNPs as biallelic markers. The results for the significance are shown in Table 1. The t-test was severely anti-conservative, nevertheless, the Mantel test behaved regularly, as for the tests by microsatellite marker. The results for power are shown in Tables 2 and 3. The power was found to be extremely poor. In fact, a trait with 50% heritability could not be detected even when a 0.01 cM map was used. This low power likely come from the poor performance of inference of coancestry by biallelic markers which have little information content. To overcome this limitation, we assumed the usage of 10 SNP haplotypes, each consisting of 10 contiguous SNPs. The FPL method was conducted by scoring a SNP haplotype as an allele. The results are also shown in Tables 2 and 3. The power was improved dramatically; it was greater than that obtained from a 1 cM microsatellite map, when a 0.01 cM SNP map was used. In fact, by additional simulations it was found that the FPL method had power larger than 80% to detect a trait with 10% heritability by 500 extremely sampled individuals from a population within a domain 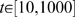, 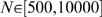, in which probably any real human population would be included. However, a trait with 5% heritability could not be detected by samples from a populations with 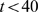 or 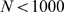.

It is important to note that the results obtained for specific parameters, as presented here, does not provide any general rule regarding the power. The parameter sets considered here were chosen solely as an illustration. The issue of power should be carefully considered for each specific study design, depending on the population demographic history, sample size, marker spacing, properties of the marker and trait locus, etc. (see Discussion).

### Bias Estimate of 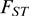; Power and Significance are Insensitive to the Bias

The estimate of 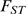 was biased, especially when a number of allelic types is small (see Appendix S1, Figure S1). This bias comes from the nature of the coancestry estimate by using genetic markers. There is possibility that, at the time when a population was founded, some of the identities of alleles may reflect independent origin (identical in state) rather than coancestry [6]. In principle, it is impossible to distinguish IBD from the identities by state by genetic markers, and the identities of alleles are always regarded as IBD. This bias was common in other estimators which we devised (data not shown). It seems that the estimation by markers with 10 different alleles still has significant bias. However, we assumed here that 10 allelic types were segregated in the founder population, since we did not want to put so restrictive assumption on polymorphism of markers.

Since the estimate of 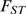 gives the prior information for the FPL method (see Methods), inaccurate estimates may influence the performance. To investigate this issue, we conducted the FPL method using the value of 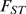 a priori. For each case of population demography, 100 populations with microsatellite maps with 10 cM interval were generated. From each simulated population 500 individuals were randomly sampled, and p-values were computed using the Mantel test. We substituted specific values of 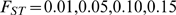 and 0.20 into Equation 1. We determined the proportion of populations for which the p-value was less than 0.05 at the trait locus of 50% heritability. We found that the power was insensitive to the substituted values of 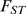. The deviations of power observed were 4%, 4%, 5%, and 8%, for Case 1–4, respectively. The significance was also found to be insensitive to the substituted values of 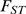 (data not shown). Thus, the FPL method will be robust against inaccurate estimate of 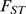. The power is sensitive to the actual values of 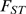.

### Population Stratification

With the FPL method, it is expected that only a few false positives may be triggered by population stratification, since the mapping method relies not on the allele frequencies but on the IBD status between individuals, as other linkage-based approaches do. However, the robustness of the FPL method against population stratification is not trivial, since the estimate of coancestry depends on the allele frequencies. We considered the robustness by examining a model of population amalgamation, in which the founder population consisted of two equal sized populations with no allelic types of markers being shared between them. We set the trait to be unlinked. For each case of population demography, we simulated 1,000 populations with microsatellite maps of 1 cM interval. From each simulated population 100 individuals were randomly sampled, and p-values were computed by the Mantel test. The proportion of the populations for which the p-value smaller than 0.05 was 0.043, 0.047, 0.046, and 0.043, for Case 1–4, respectively. Inflate of the significance by population stratification seems to be absent.

### Allelic Heterogeneity

The properties of the trait locus affect power of the FPL method. We considered the model where two alleles are segregating in the trait locus in the founder population (we will call this the single segregating site model). Here, two other models of allelic heterogeneity in the trait locus were considered. First, we considered the ten segregating sites model, whereby the ten sites have an equal effect and are segregating in the founder population. Compared to the single segregating site model, the ten segregating sites model exhibited no significant power reduction (data not shown). The second model was the infinite sites mutation model with a mutation rate of 0.001 for each site and mutation should occur at a non-segregating site. We assumed that none of the mutations existed in the founder population and that all the segregating sites have equal effects. Under the infinite site mutation model, a large number of low frequency alleles appear and the locus becomes highly heterogeneous. We set the heritability to be 50%, and the additive model was assumed. For each case of population demography, we simulated 100 populations with microsatellite maps of 1 cM interval. From each population 500 individuals were sampled randomly, and p-values were computed by the Mantel test. The proportions of the populations for which the p-value smaller than 0.05 at the trait locus were 33%, 93%, 33%, and 84%, for Case 1–4, respectively. Compared to the single segregating site model, the infinite site mutation model exhibited a significant power reduction.

### Comparison with standard pedigree-based linkage analysis

**Figure 3:**
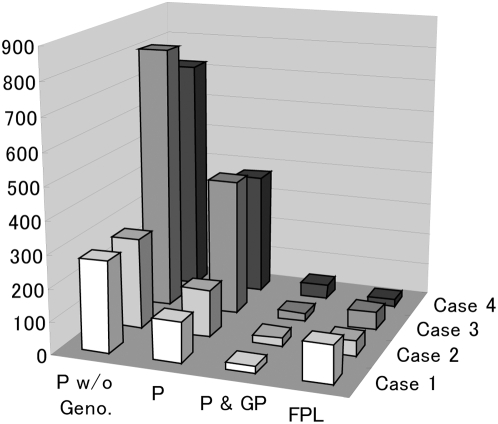
The numbers of individuals needed to detect the trait locus with 80% power with each scenario by the FPL method and by a standard pedigree-based linkage mapping method (SOLAR). Four cases of population demography, Case 1–4 are simulated (see Method). Four scenarios are assumed: 1) genotypes of sample are analyzed with the FPL method (FPL); 2) genotypes of sample with the complete pedigree up to the parental generation are analyzed with SOLAR (P w/o Geno.); 3) genotypes of sample and their parents and the complete pedigree up to the parental generation are analyzed with SOLAR (P); 4) genotypes of sample, their parents, and their grand parents, and the complete pedigree up to the grand parental generation are analyzed with SOLAR (P & GP).

Using simulations, we compared the power of the FPL method with that of a standard pedigree-based linkage mapping method, which is known as the variance component approach and implemented in the SOLAR computer software package [14]. Currently SOLAR is probably the most popular program for identification of quantitative trait loci by standard pedigree-based approach. For each case of population demography, we computed number of randomly sampled individuals needed to detect an additive trait locus with 100% heritability by microsatellites maps with 0 cM intervals. Four scenarios are assumed: 1) genotypes of samples are analyzed with the FPL method; 2) genotypes of samples with the complete pedigree up to the parental generation are analyzed with SOLAR; 3) genotypes of samples and their parents, and the complete pedigree up to the parental generation are analyzed with SOLAR; 4) genotypes of sample, their parents, and their grand parents, and the complete pedigree up to the grand parental generation are analyzed with SOLAR. Note that we did not used the parental genotypes for the FPL method. The numbers of individuals required to detect the trait locus with 80% power are shown in Figure 3. It can be seen that the FPL method would be generally powerful than SOLAR even if genotype of parents of sample are used. When we use three-generation genotypes and pedigrees, namely in the fourth scenario, SOLAR could be more powerful than the FPL method.

### Results Obtained for the Actual Data

To demonstrate the applicability of the FPL method, we determined the genotypes of 8 microsatellite markers on the chromosome 6 of 375 Japanese patients with psoriasis vulgaris and 375 healthy controls. All the participants are unrelated according to pedigree records. The average marker spacing was 10.4 cM. A marker D6S2931 is located in PSORS1 at 6q21.3 near the HLA-C gene, which has been shown to be significantly linked and associated with psoriasis [15]. The susceptible allele of D6S2931 was 379 (p = 0.000008; Fisher's exact test). Recently, a genome-wide scan reveals that a SNP rs12191877, which is 13 kb upstream of the HLA-C gene, shows strong association to psoriasis in European and is in linkage disequilibrium with the HLA-Cw6 haplotype [16].

First, using the 375 healthy controls, we estimated coancestry for these markers. The distribution of 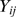 did not differ significantly among the markers (data not shown); this result supports the notion that the Japanese demographic history is a major factor having shaped the distribution. Modern humans are considered to have entered the Japanese islands around 30,000 years ago, when the Japanese islands were connected with continental Asia. However, the Japanese islands became completely disconnected from continental Asia about 12,000 years ago; this implies that the Japanese have probably been isolated for about 12,000–30,000 years. There were occasional migratory waves from continental Asia (a debate on the influence of these migrations with reference to several aspects, including molecular data, is reviewed by [17]). Recently, studies conducted using whole genome SNPs data of Japanese people showed that 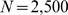 [18] and 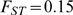 [7]. According to these estimates, we have 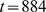, which is consistent with the estimate of the isolation period mentioned above (17,680 years if we assume a generation time is 20 years). Figure S2 shows comparison of the observed and simulated distribution of estimates of coancestry.

**Figure 4:**
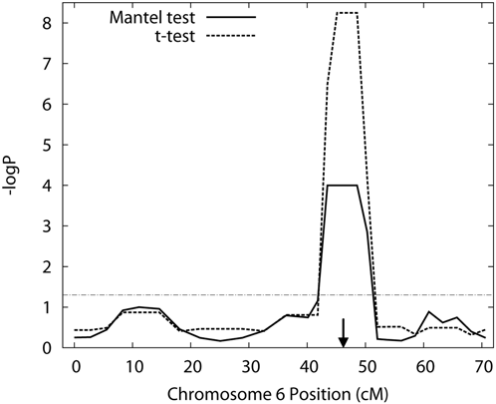
Plot of p-values obtained by the FPL method for psoriasis on the chromosome 6 using 750 unrelated Japanese individuals. The horizontal line represents the 0.05 level, and the arrow represents the position of the marker D6S2931 which is located in the PSORS1 locus. 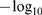 of the p-values by the Mantel test and the t-test of the regression are shown.

Subsequently, using the 375 healthy controls and the 375 psoriasis patients (750 individuals in total), we conducted linkage mapping by using the new method. Figure 4 shows the p-values for the linkage signals by the Mantel test and the t-test of the regression; in the Mantel test, the strongest signal was detected at the position of D6S2931 (p = 0.0001). Apart from the linkage detected for the 7 cM interval surrounding D6S2931, no significant linkage was detected at the 5% significance level. When D6S2931 was dropped from the data set, no significant linkage was detected. The computation of the p-values on a 2.66-GHz Xeon processor, was completed within 25 min. Our results met our expectations in light of the fact that D6S2931 is only 30 kbp upstream from the susceptible gene HLA-C. Our results do not provide any evidence of 10.4 cM intervals between markers being sufficient for detecting the linkage. However, according to our simulation, the power of the mapping was probably adequate, since its value with extreme sampling was 74% for additive trait locus with 50% heritability.

## Discussion

In the present paper, we presented a new linkage mapping method within a finite population under random mating, without pedigree records. In addition, we assessed the feasibility of this mapping by using simulated and actual data. This method is based on the estimation of coancestry for a chromosomal position between individuals, with the use of multipoint Bayesian estimation. Association-based approaches are commonly used for unrelated individuals, when it is difficult to obtain an extended pedigree for a population. We hope that our linkage mapping method may provide a basis for linkage mapping approach of quantitative trait within such populations, since linkage-based approaches have several advantages when compared to association-based approaches. First, the linkage-based approaches remain powerful even in case of allelic heterogeneity. Second, only a few false positives are triggered by population stratification, since these approaches rely not on the allele frequency but on the IBD status. Third, as mentioned above, the significance of the new linkage mapping method does not increase with population stratification, and its power is retained in cases of mild allelic heterogeneity. Finally, in contrast to standard pedigree-based linkage analysis, the new linkage mapping method described here is robust against the increase in type I errors caused by linkage disequilibrium among markers.

Remarkably, for random samples the FPL method would be generally more powerful than a standard pedigree-based analysis, even if we additionally determine genotypes of parents of sample for the pedigree-based analysis. It might seem counter-intuitive that the FPL method is powerful than a standard pedigree-based linkage analysis in the respect of sample size, but it is a reasonable consequence because we assumed that we correct the sample without caring the pedigrees. Standard pedigree-based linkage analyses use known kinship among individuals, but such close kinship is rare in randomly sampled individuals. In contrast, the FPL method uses all kinship, which might be remote but would be in abundance within a finite population. Another interesting question is how extensive pedigree information is needed for standard pedigree-based linkage approach to over perform the FPL method, when we do not know genotypes of the ancestors. Unfortunately, pedigree-based linkage analyses are so computer intensive that we could not address this issue, but three generation pedigree information was not enough to over perform the FPL method.

Understanding the relatedness between individuals is important for many aspects of genetics and ecology. Many different estimators have been developed for kinship coefficient in infinitely large populations [19]–[21]; however these estimators are designed to capture nonrandom inbreeding caused by consanguineous marriages within an infinitely large population. On the other hand, the new linkage mapping method presented in the present paper uses random inbreeding within a finite population, which can be measured in terms of Wright's 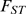. Recently, [22] discussed usage of unlinked SNPs which are genotyped for association scans to detect linkage with nonrandom inbreeding caused by consanguineous marriages within an infinitely large population, without pedigree records. The method is apparently similar to the new linkage mapping method presented in the present article, in the sense that both of the methods do not need pedigree records. However, types of data to which the methods should apply are different. Also, the practical utility of the method proposed by [22] is still unknown, because the paper does not study practical issues such as the significance and power of their approach and its applicability to actual data.

The new linkage mapping method in itself does not require any of the parameters detailing population demographic history. However, the power of the new linkage mapping method is a complicated function of these parameters. For example, the power is not necessarily gained by use of a population with high 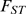 values. High 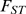 values are expected in a small sized old population; however, in an old population, it is difficult to detect linkage by using loosely linked markers, since a large numbers of recombinations accumulated along the genealogy of the sample. Thus, for assessing the power of the new linkage mapping method, it is necessary to have a rough idea of the population demographic history of the concerned population. They can be estimated on the basis of the genotypes determined for a particular region of genomes of a small sample. By comparing the observed and simulated distributions of estimates of coancestry using various parameter sets, we could choose parameters which are suitable for the researchers own sample (see Results). Subsequently, by performing a simulation using the estimated parameters, we could compute the power of the method in relation to the sample size, the marker density, and the heritability of the trait locus. As an illustration, assume we have an extreme sample of 500 Japanese individuals and we want to detect a locus with 10% heritability. We have estimated that the Japanese population founded 884 generations ago and the effective size is 2,500. According to our simulations, considering microsatellite markers with 1 cM spacing, the estimated power would be 99% and 86%, when the additive and completely dominant models for the trait locus, respectively. However, with 5 cM marker spacing, these respective power values would be 32% and 20%. Thus, marker spacing of 1 cM should be sufficient for detecting the trait locus, and a resolution of less than 5 cM could be achieved. With SNP haplotypes consists of SNP markers spaced every 0.1 cM, the estimated power were 100% and 98%, for additive and completely dominant model, respectively. Since the new linkage mapping method and the procedure for assessing its power are computer intensive, we have developed a computer software package named FPL (Finite Population based Linkage mapping method), which is available upon request.

## Supporting Information

Appendix S1 (0.23 MB DOC)

Table S1 (0.11 MB DOC)

Table S2 (0.06 MB XLS)

Figure S1 The moment estimates of Fst as a function of number of allelic types. The horizontal lines show the actual values of Fst. This figure shows bias, especially for small number of alleles.
(1.12 MB TIF)

Figure S2 Posterior expectation of coancestry (see Methods), which is the expected proportion of the number of shared alleles of a marker between a pair of individuals by IBD. Results obtained by real data of a marker D6S962 of 375 unrelated healthy Japanese individuals and by those of simulated data are shown. For the simulations we assumed that the Japanese population founded 884 generations ago and the effective size is 2,500. We simulated 10 populations with these parameters (10×375×374/2 = 701250 pairs in total). The left ordinate is for the real data and the right ordinate is for the simulated data. Although simulated and observed distributions were similar, they differed to some degree. It probably means that the demography of the Japanese is not as simple as was assumed in the isolated random mating model.
(1.35 MB TIF)
