# Supplementary material for: Detecting Linkage between a Trait and a Marker in a Random Mating Population without Pedigree Record
Source: PLoS One. 2009 Mar 24;4(3):e4956. doi: 10.1371/journal.pone.0004956 (PMC2655708; doi:10.1371/journal.pone.0004956)
Supplement: Appendix S1 — (0.23 MB DOC) [file pone.0004956.s001.doc]

Appendix S1

Shuhei Mano et al., Detecting linkage between a trait and a marker in a random mating population without pedigree record

Correspondence: E-mail: mano@nsc.nagoya-cu.ac.jp

**Probability distribution of** $X_{ij}$

Consider a random mating population that comprises of $N$ diploid individuals and was founded $t$ generations ago. Denote coalescence time to the most recent common ancestor of $k$ alleles randomly sampled from a population by $T_{k}$. Since $T_{k}\sim Exp(k(k-1)/4N)$, $P(T_{4}>s)=\exp(-6s/2N)$. By the Markov property, we have

$P(X_{ij}=1)=P(T_{3}+T_{4}>s>T_{4})=\int_{0}^{s} dv\int_{s-v}^{\infty} du\frac{6}{2N}e^{-6v/2N}\frac{3}{2N}e^{-3u/2N}=2(e^{-3s/2N}-e^{-6s/2N})$.

In the same manner,

$P(X_{ij}=6)=P(s>T_{2}+T_{3}+T_{4})=1-\frac{1}{5}(9e^{-s/2N}-5e^{-3s/2N}+e^{-6s/2N})$.

When two coalescence events occur in $(0,t)$, the genealogy exhibits one of the two possible topologies of the genealogy. The probability that the third topology in Figure 1 appears is 1/3 and that the fourth topology appears is 2/3. Thus,

$P(X_{ij}=2)=\frac{1}{2}P(X_{ij}=3)=\frac{1}{3}P(T_{2}+T_{3}+T_{4}>s>T_{3}+T_{4})=\frac{1}{5}(3e^{-s/2N}-5e^{-3s/2N}+2e^{-6s/2N})$.Note that $P(X_{ij})$ is determined solely by $F_{st}$, since $e^{-t/2N}=1-F_{ST}$.

**Derivation of Equation 1**

We have

$P(G_{ij},Y_{ij})=\sum_{S_{ij}\subset Y_{ij}} P(G_{ij}|S_{ij},Y_{ij})P(S_{ij},Y_{ij})=\sum_{S_{ij}\subset Y_{ij}} P(G_{ij}|S_{ij})\sum_{X_{ij}} P(S_{ij}|X_{ij},Y_{ij})P(X_{ij},Y_{ij})$.

Dividing both hand sides of the equation by $P(Y_{ij})$, we obtain Equation 1. It is straightforward to obtain $P(S_{ij}|X_{ij},Y_{ij})$ by the definition of $S_{ij}$ (see Method). For example, $P(S_{ij}=5|X_{ij}=3,Y_{ij}=1)=1/2$, since $X_{ij}=3,Y_{ij}=1$ is identical to a union of mutually exclusive and equally probable events: $S_{ij}=3$ and $S_{ij}=5$. It can be seen from Figure 2 that

$P(Y_{ij}=0|X_{ij}=1)=P(Y_{ij}=0|X_{ij}=2)=1/3$,

$P(Y_{ij}=1|X_{ij}=1)=P(Y_{ij}=2|X_{ij}=2)=2/3$,

$P(Y_{ij}=0|X_{ij}=0)=P(Y_{ij}=1|X_{ij}=3)=P(Y_{ij}=2|X_{ij}=6)=1$.

Thus, we have$P(X_{ij},Y_{ij})=P(Y_{ij}|X_{ij})P(X_{ij})$ and $P(Y_{ij})=\sum_{X_{ij}} P(X_{ij},Y_{ij})$. Since $P(X_{ij})$ is determined by $F_{st}$, $P(G_{ij}|Y_{ij})$ depends on $F_{st}$. In addition, $P(G_{ij}|Y_{ij})$ depends on allele frequencies of markers since $P(G_{ij}|S_{ij})$ in Table 1 depends on the allele frequencies.

**Computation of multipoint posterior probability distribution of** $Y_{ij}$

. The chain of $Y_{ij},j=1,2,...,m$ is characterized by a multipoint coalescence genealogy, the ancestral recombination graph [A1]. It is known that the process to construct the ancestral recombination graph when moving spatially along a chromosome is non-Markovian [A2], however, we assume the chain of $Y_{ij}$ to be Markovian such that we can apply the hidden Markov model [A3]. Simulation with moving in time in Method accounts for the non-Markovian nature when we see the process with moving spatially [A1-A3]. By the simulation with moving in time, it was found that the Markov assumption for the chain of $Y_{ij}$ to a satisfactory degree (data not shown). $Y_{ij}$ can be modeled by a three-state hidden Markov model, where $Y_{ij}$ are the hidden states and the genotypes are the symbols. Similar hidden Markov models have been used for association and linkage mapping methods [A4-A7]. The emission probability has already been computed (Equation 1). In practice, we replace $P(G_{ij}|S_{ij})$ for $S_{ij}\neq9$ in Table 1 with to allow for genotyping errors and mutation with the error parameter $\varepsilon$ (we set $\varepsilon=0.01$) [A6]. Further, we set for missing genotypes, which amounts to assuming the missing genotype mechanism is independent of the IBD mode [A6]. For $F_{ST}$, we use the average of the estimates for each marker. Let the transition probability be $t_{uv}=P(Y_{i,j+1}=u|Y_{ij}=v)$, where a common value is assumed for all intervals between the markers (relaxation of the assumption will be discussed below). The maximum likelihood estimates of the transition probabilities can be computed by using the Baum-Welch algorithm [A8]. The transition probability matrix is 3-dimensional and the results did not significantly depend on the initial value (data not shown). To avoid over fitting, we place pseudo counts following $P(Y_{ij})$ whose size is the square root of the observed counts [A8]. When marker spacing is significantly uneven, a common transition probability for all intervals between the markers will not be adequate. To take account of unevenness in the marker spacing, we employ a variable model for the transition probability:$t_{uv}=\delta_{uv}e^{-ag_{j,j+1}}+P(Y_{ij}=v)(1-e^{-ag_{j,j+1}})$, where $g_{j,j+1}$ is the genetic distance between the markers, $a$is roughly the time to the most recent common ancestor [A6], and$\delta_{uv}$ is the Kronecker’s delta. This model is an extension of the model of IBD probability of two homologous genes of a single individual [A6]. The least square estimate of $a$ is obtained by using the maximum likelihood estimates of $t_{uv}$ and the average marker spacing. Then, we computed the multipoint posterior probability distribution $P(Y_{ij}|G_{i1},G_{i2},...,G_{im})$ by using the forward and backward algorithm [A8].

**Conditional expectation of** $Z_{i}^{2}$

As [A9] showed and extensions to general relative pairs were discussed by [A10], the linkage between a trait and a marker is detected for each marker by estimating the number of alleles shared IBD between pairs of individuals for each marker. Let the absolute differences between the trait values for the $i$-th pair of individuals be $Z_{i}$. By using variance and covariance of trait values among individuals within an inbred population [A11,A12], the expectation of squared difference of trait values between a pair of individuals randomly chosen from a population is $E(Z_{i}^{2})=(2+2f_{i}-4\Phi_{i})V_{a}+2V{}_{e}+\cdots$, where dots represent terms that vanish without dominance. $f_{i}$ is the kinship coefficient for the two individuals, $\Phi_{i}$ is the inbreeding coefficients between the$i$-th pair of individuals, and $V_{a},V_{e}$ are the additive variance and the environmental variance of the trait, respectively. In a random mating population, $f_{i}=\Phi_{i}=P(Y_{ij}=1)/4+P(Y_{ij}=2)/2+P(X_{ij}=3)/4+P(X{}_{ij}=6)/2$. Here, the expression is given solely by $F_{ST}$ and the last two terms are $O(F_{ST}^{2})$ (see Derivation of Equation 1). When $Y_{ij}$ is given, the conditional expectation is $E(Z_{i}^{2}|Y_{ij})=2(V_{a}+V_{e})-V_{a}Y_{ij}/2+\cdots$ with ignoring terms $O(F_{ST}^{2})$.

**The Mantel test**

Our aim is to detect an association between $Z_{i}$, which represents the absolute difference between trait values for the $i$-th pair, and the multipoint posterior estimate of $Y_{ij}$, which is the estimated proportion of alleles shared IBD between the $i$-th pair of individuals at the $j$-th marker. Our task is to detect association between the two symmetric dissimilarity matrices $Z_{i}$ and $E(1-Y_{ij}/2|G_{i1},G_{i2},...,G_{im})$. The Mantel test detects the association between two independent dissimilarity matrices describing a set of entities and assesses whether the association is stronger than one would be expected by chance [A13]. The Mantel statistic is the Hadamard product of the two dissimilarity matrices. To test whether the statistic is a significantly deviant value, we can carry out a randomization test. We implement a sampled permutation test, in which the elements of one matrix are randomly altered by permutating the rows (or columns). We randomly permutate the elements of one of the matrices, calculating the statistic for the randomized values each time. Subsequently, we are able to evaluate the probability of the observed value of the statistic by noting its position in the distribution of randomized outcomes.

**The interval mapping**

An interval mapping method can give an estimate of coancestry corresponding to $Y_{ij}$ at arbitrary points on a map [A14]. Assume we have . Let represents the coancestry at a position which lies between the $j$-th and the $j+1$-th markers. Assume a regression equation $\Pi_{iq}=\alpha+\beta_{j}\Pi_{ij}+\beta_{j+1}\Pi_{ij+1}$. $Cov[\Pi_{ij},\Pi_{iq}]$ and $Cov[\Pi_{i,j+1},\Pi_{iq}]$ are linear combinations of $\beta_{j}$ and $\beta_{j+1}$, where $E[\Pi_{ij}]=E[Y_{ij}]/2,V[\Pi_{ij}]=V[Y_{ij}]/4$, and $4Cov[\Pi_{ij},\Pi_{iq}]=\sum_{u,v} uvt_{uv}P[Y_{ij}=v]-E[Y_{ij}]^{2}$ [A14]. To obtain the value of $Cov[\Pi_{ij},\Pi_{iq}]$, we may assume $Cov[\Pi_{ij},\Pi_{iq}]=V[\Pi_{ij}]e^{-bg_{jq}}$, where $b$ is the decay rate of the covariance and $g_{jq}$ is the genetic distance between the positions $j$ and $q$. It is possible to estimate $b$ by solving $Cov[\Pi_{ij},\Pi_{i,j+1}]=V[\Pi_{ij}]e^{-bg_{j,j+1}}$ for $b$. Then, $\beta_{j},\beta_{j+1}$ and $\alpha$ can be estimated by solving the equations for $Cov[\Pi_{ij},\Pi_{iq}]$ and $Cov[\Pi_{i,j+1},\Pi_{iq}]$. Substituting these values into the regression equation, we can estimate via and . is a regression estimates based solely on the estimates of coancestry at two nearest neighboring markers, nevertheless, the estimates of coancestry at the two nearest neighbor markers are based on the full likelihood which is derived from the information of all the markers.

**Estimation of** $F_{ST}$

For Case 1 and 2 of population demographies, we simulated 100 populations with microsatellite maps with 1 cM interval. From each simulated population 500 individuals were randomly sampled and $F_{ST}$ was estimated (see Methods). For each marker, the number of different alleles in the founder population was set to be 2, 10, 20, 30, 40, and 50. The actual number of allelic types could be smaller than these figures, since some of the allelic types could be extinct by random drift. The results are shown in Figure S1. It shows clear bias, especially for small number of alleles.

**References**

[A1] Griffiths RC, Majoram P (1997) An ancestral recombination graph. In: Donnely P and Tavare S, editors. Progress in population genetics and human evolution. IMA volumes in mathematics and its applications, Vol. 87, Springer-Verlag, Berlin. pp. 257-270.

[A2] Wiuf C, Hein J (1999) Recombination as a point process along sequences. Theor Popul Biol 55: 248-259.

[A3] Cardin, McVean (2005) Approximating the coalescent with recombination. Philos Trans R Soc Lond B Biol Sci 360: 1387-1393.

[A4] McPeek MS, Strahs A (1999) Assessment of linkage disequilibrium by the decay of haplotype sharing, with application to fine-scale genetic mapping. Am J Hum Genet 65: 858-875.

[A5] Morris AP, Whittaker JC, Balding DJ (2000) Bayesian fine-scale mapping of disease loci, by hidden markov models. Am J Hum Genet 67: 155-169.

[A6] Abney M, Ober C, McPeek MS (2002) Quantitative-trait homozygosity and association mapping and empirical genomewide significance in large, complex pedigrees: fasting serum-insulin level in the Hutterites. Am J Hum Genet 70: 920-934.

[A7] Leutenegger AL, Prum B, Genin E, Verny C, Lemainque A, et al. (2003) Estimation of the inbreeding coefficient through use of genomic data. Am J Hum Genet 73: 516-523.

[A8] Durbin R, Eddy SR, Krogh A, Mitchison G, Biological Sequence Analysis: Probabilistic Models of Proteins and Nucleic Acids. Cambridge University Press, Cambridge.

[A9] Haseman JK, Elston RC (1972) The investigation of linkage between a quantitative trait and a marker locus. Behav Genet 2: 3-19.

[A10] Olson JM, Wijsman EM (1993) Linkage between quantitative trait and marker loci: methods using all relative pairs. Genet Epidemiol 10: 87-102.

[A11] Harris DL (1964) Genotypic covariances between inbred relatives. Genetics 50: 1319-1348.

[A12] Abney M, McPeek MS, Ober C, 2000. Estimation of variance components of quantitative traits in inbred populations. Am J Hum Genet 66: 629-650.

[A13] Sokal RR, Rohlf FJ (1995) Biometry 3d ed. WH Freeman and Company, New York.

[A14] Fulker DW, Cardon LR (1994) A sib-pair approach to interval mapping of quantitative trait loci. Am J Hum Genet 54: 1092-1103.
