# Supplementary material for: Detecting Linkage between a Trait and a Marker in a Random Mating Population without Pedigree Record
Source: PLoS One. 2009 Mar 24;4(3):e4956. doi: 10.1371/journal.pone.0004956 (PMC2655708; doi:10.1371/journal.pone.0004956)
Supplement: Table S1 — (0.11 MB DOC) [file pone.0004956.s002.doc]

Table S1. Conditional probability of genotypes given modes of identity by descent (IBD)

| Nine IBD modes ($S$) | | | | | | | | | |
| --- | --- | --- | --- | --- | --- | --- | --- | --- | --- |
| Genotypes | 1 | 2 | 3 | 4 | 5 | 6 | 7 | 8 | 9 |
| $ii,ii$ | $p$ | $p^{2}$ | $p^{2}$ | $p^{3}$ | $p^{2}$ | $p^{3}$ | $p^{2}$ | $p^{3}$ | $p^{4}$ |
| $ii,jj$ | 0 | $pq$ | 0 | $pq^{2}$ | 0 | $p^{2}q$ | 0 | 0 | $p^{2}q^{2}$ |
| $ii,ij$ | 0 | 0 | $pq$ | $2p^{2}q$ | 0 | 0 | 0 | $p^{2}q$ | $2p^{3}q$ |
| $ii,jk$ | 0 | 0 | 0 | $2pqr$ | 0 | 0 | 0 | 0 | $2p^{2}qr$ |
| $ij,ii$ | 0 | 0 | 0 | 0 | $pq$ | $2p^{2}q$ | 0 | $p^{2}q$ | $2p^{3}q$ |
| $jk,ii$ | 0 | 0 | 0 | 0 | 0 | $2pqr$ | 0 | 0 | $2p^{2}qr$ |
| $ij,ij$ | 0 | 0 | 0 | 0 | 0 | 0 | $2pq$ | $pq(p+q)$ | $4p^{2}q^{2}$ |
| $ij,ik$ | 0 | 0 | 0 | 0 | 0 | 0 | 0 | $pqr$ | $4p^{2}qr$ |
| $ij,kl$ | 0 | 0 | 0 | 0 | 0 | 0 | 0 | 0 | $4pqrs$ |

Deferent allelic types are denoted by $i,j,k,l$. Frequencies of the alleles $i,j,k,l$ are $p,q,r,s,$respectively.
