# Supplementary figures and images for: Detecting Linkage between a Trait and a Marker in a Random Mating Population without Pedigree Record
Source: PLoS One. 2009 Mar 24;4(3):e4956. doi: 10.1371/journal.pone.0004956 (PMC2655708; doi:10.1371/journal.pone.0004956)

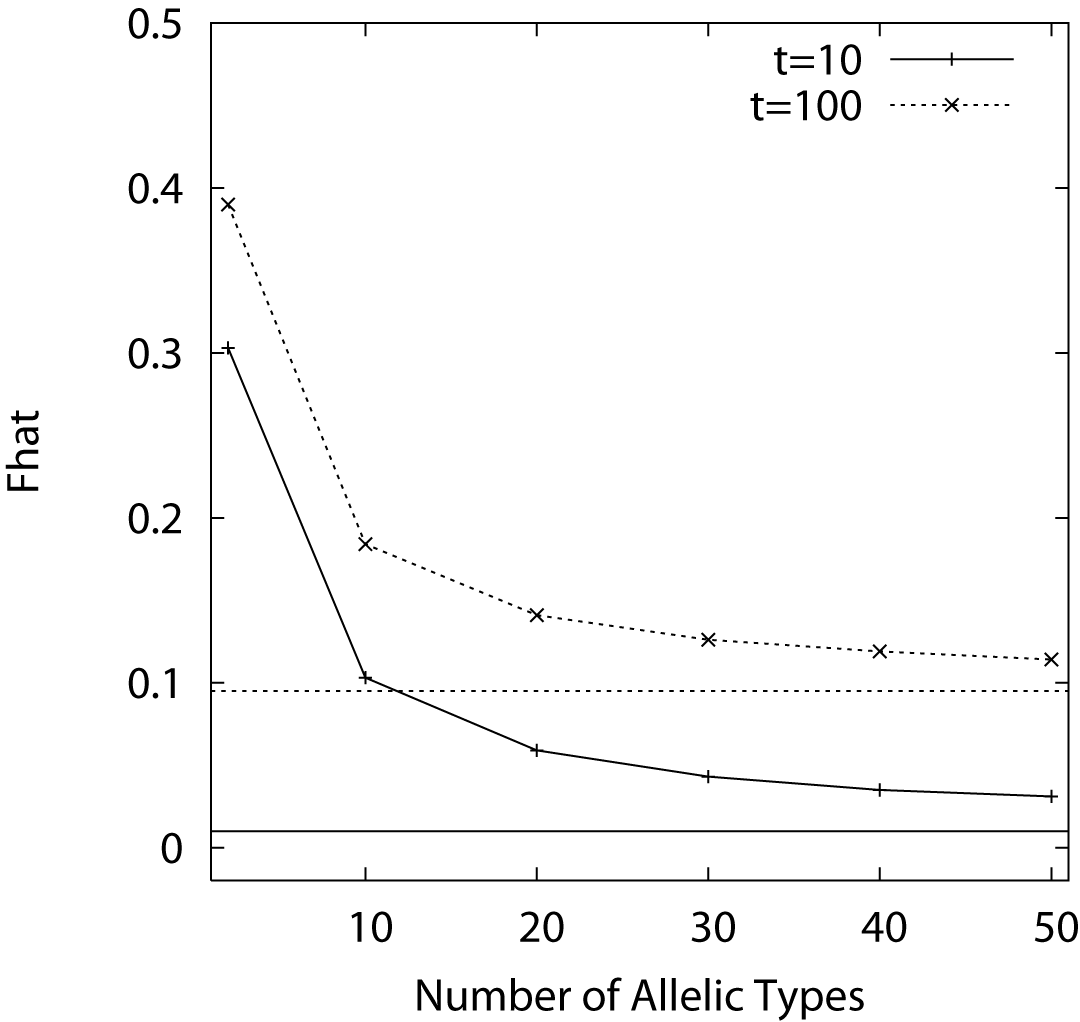

Supplement: Figure S1 — The moment estimates of Fst as a function of number of allelic types. The horizontal lines show the actual values of Fst. This figure shows bias, especially for small number of alleles. (1.12 MB TIF) [file pone.0004956.s004.tif]

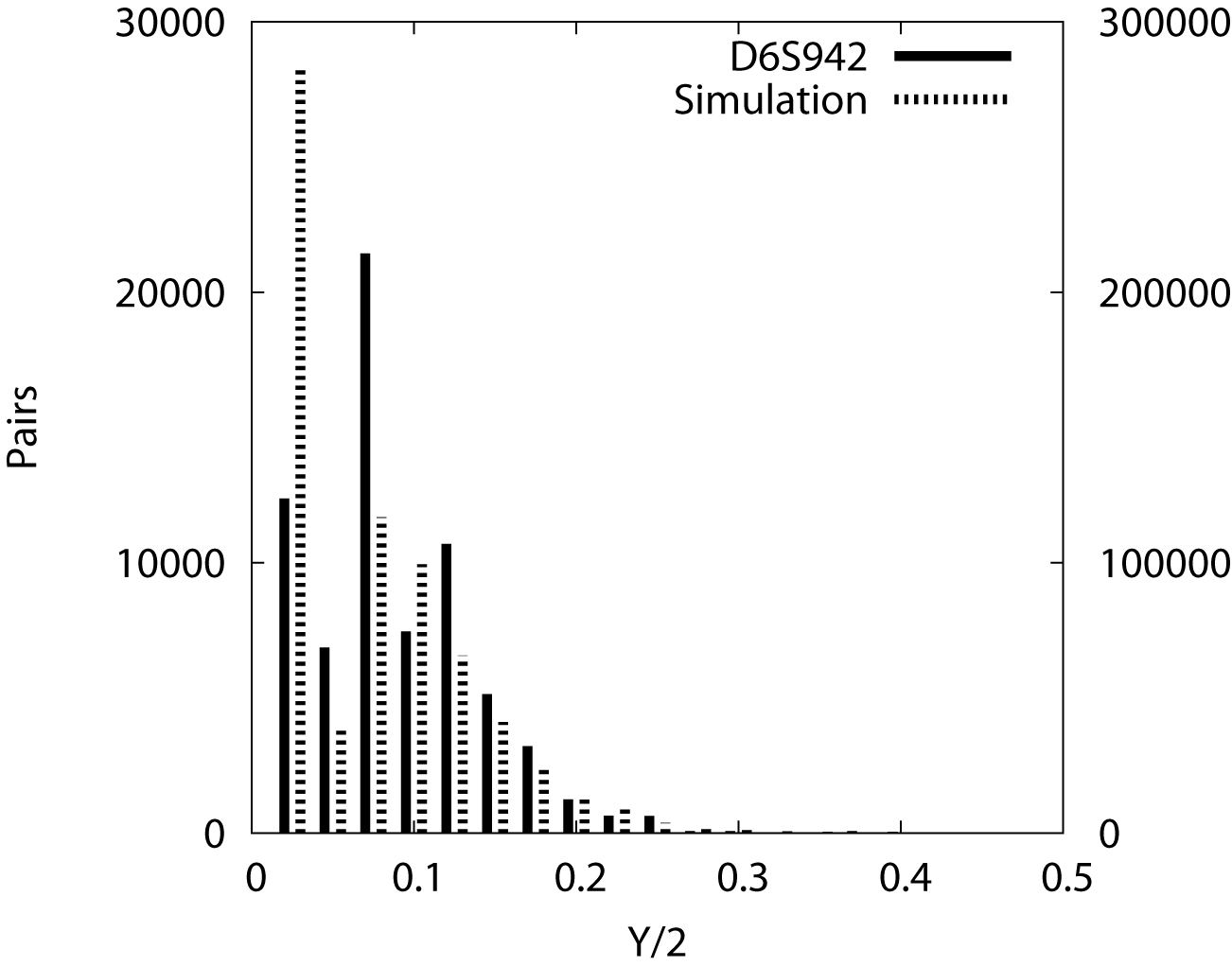

Supplement: Figure S2 — Posterior expectation of coancestry (see Methods), which is the expected proportion of the number of shared alleles of a marker between a pair of individuals by IBD. Results obtained by real data of a marker D6S962 of 375 unrelated healthy Japanese individuals and by those of simulated data are shown. For the simulations we assumed that the Japanese population founded 884 generations ago and the effective size is 2,500. We simulated 10 populations with these parameters (10×375×374/2 = 701250 pairs in total). The left ordinate is for the real data and the right ordinate is for the simulated data. Although simulated and observed distributions were similar, they differed to some degree. It probably means that the demography of the Japanese is not as simple as was assumed in the isolated random mating model. (1.35 MB TIF) [file pone.0004956.s005.tif]
